# Test–Retest Reliability of Task-Oriented Strength and Object Position in a Box Lifting Task Using the Activities of Daily Living Test and Training Device (ADL-TTD) in Children with Unilateral Spastic Cerebral Palsy

**DOI:** 10.3390/children12081030

**Published:** 2025-08-05

**Authors:** Haowei Guo, Inge Heus, Bart Snijders, Nanne E. Land, Menno van der Holst, Rob. J. E. M. Smeets, Caroline H. G. Bastiaenen, Eugene A. A. Rameckers

**Affiliations:** 1 Department of Rehabilitation Medicine, Research School CAPHRI, Maastricht University, 6229 ER Maastricht, The Netherlands; r.smeets@maastrichtuniversity.nl (R.J.E.M.S.); eugene.rameckers@maastrichtuniversity.nl (E.A.A.R.); 2 Center of Expertise, Adelante Pediatric Rehabilitation, 6432 CC Hoensbroek, The Netherlands; 3 Research Centre for Assistive Technology in Health Care, Zuyd University of Applied Sciences, 6419 PB Heerlen, The Netherlands; inge.heus@zuyd.nl; 4 Department of Pediatric Rehabilitation, Revant Rehabilitation Centers, 4817 JW Breda, The Netherlands; b.snijders@revant.nl; 5 Center for Rehabilitation ‘Revalidatie Friesland’, 9244 CL Friesland, The Netherlands; n.land@revalidatie-friesland.nl; 6 Department of Orthopaedics, Rehabilitation and Physiotherapy, Leiden University Medical Center, 2333 ZG Leiden, The Netherlands; me.van.der.holst@basaltrevalidatie.nl; 7 Department of Innovation, Quality & Research, Basalt Rehabilitation the Hague, 2543 SW Den Haag, The Netherlands; 8 CIR Clinics in Revalidatie, 5628 WB Eindhoven, The Netherlands; 9 Department of Epidemiology, Research Line Functioning, Rehabilitation and Participation CAPHRI, Maastricht University, 6229 ER Maastricht, The Netherlands; chg.bastiaenen@maastrichtuniversity.nl; 10 Rehabilitation Science and Physiotherapy, Hasselt University, 3590 Diepenbeek, Belgium

**Keywords:** cerebral palsy, muscle strength, positional performance, test–retest reliability, upper extremity, activity of daily living, task-oriented, technology

## Abstract

**Purpose**: This study investigates the test–retest reliability of maximal voluntary contraction (MVC) and integrated object positioning during bimanual box lifting tasks in children with unilateral spastic cerebral palsy (USCP), using the Activities of Daily Living Test and Training Device (ADL-TTD). **Materials and Methods**: Utilizing an explorative cross-sectional design, the study recruited 47 children with USCP. The ADL-TTD, equipped with an Inertial Measurement Unit (IMU) for precise object positioning, measured MVC, and object position in 3D space in a cross-sectional measurement containing two measurements in a fixed time period. **Results**: The findings demonstrated good test–retest reliability for MVC, with an ICC_agreement_ of 0.95 for the mean MVC value. Additionally, good reliability was observed for object positioning in different directions measured with an IMU, with ICC_agreement_ ranging from 0.82 to 0.86 degrees. Regarding the standard error of measurement (SEM), the SEM_agreement_ for the mean MVC value was 5.94 kg, while the SEM_agreement_ for object positioning was 1.48, 5.39, and 3.43 degrees, respectively. **Conclusions**: These results indicate that the ADL-TTD demonstrates good test–retest reliability for both MVC and object positioning, making it a valuable tool for analyzing this population in cross-sectional research by providing reliable measures of task-oriented strength and object manipulation. However, the relatively high SEM_agreement_, particularly in MVC, suggests that caution is needed when using this tool for repeated testing over time. This pioneering approach could significantly contribute to tailored assessment and training for children with USCP, highlighting the importance of integrating task-specific strength and positional accuracy into therapeutic interventions.

## 1. Introduction

Unilateral spastic Cerebral Palsy (USCP) is a neurological disorder caused by brain injury, either during fetal development or in the first year after birth. USCP is on average present in one third of all children diagnosed with cerebral palsy [1]. USCP is characterized by motor impairments that mainly affect only one side of the body. As a result, one upper extremity (UE) is typically identified as the affected side, with the opposite UE being referred to as the non-affected side [2]. This condition presents various symptoms affecting UE functionality [3]. Specifically, children with USCP often exhibit decreased strength in their affected UE and diminished precision in fine motor tasks [4]. These symptoms invariably decrease task performance and limit activities of daily living (ADLs), such as carrying objects with both hands, eating, lacing shoes, and manipulating objects [5]. In these bimanual tasks, the non-affected side typically takes the lead, with the affected side assisting. Children with USCP often struggle with bimanual coordination, making tasks that require the use of both hands challenging [6].

Assessment of UE strength in children with USCP is commonly performed by grip and pinch strength measurements, as well as by isometric measurements of the arm muscles using hand-held dynamometry (HHD) that have moderate to good reliability in children with USCP [7]. The international guideline for Cerebral Palsy, however, advises assessment and training that is task-specific, goal-directed, and needs-based [8]. It further advises that the strength assessment should also be task-specific, goal-directed, and needs-based, which grip, pinch, and HHD strength measurements are not. Dekkers et al., 2019, presented the first task-specific strength assessments: the unimanual cup-lifting and bimanual box-lifting tasks. Strength is assessed by loading a box and cup with weights and determining the maximal voluntary contraction (MVC) in each task. The measurement of strength during the performance of both tests showed good test–retest reliability [9]. Geijen et al., 2019, developed a Task-oriented Arm–Hand Capacity (TAAC) instrument to assess task-specific strength by using a force sensor connected to a box, a cup, or a press fastener to measure the MVC while lifting a box or cup or pressing the fastener. These measures showed moderate-to-good test–retest reliability and validity [10].

Next to the MVC, task performance accuracy is very relevant because the ability to avoid spilling liquid or food from the box or cup, dropping the object, or not being able to close a fastener greatly affects the child’s ADL. Task performance has been defined by speed (temporal precision), smoothness, and spatial precision of an executed task [11]. These components can be measured by using a sensor to track the trajectory of an object in three dimensions [12]. Technology such as the Inertial Measurement Unit (IMU) can be used to measure the position of an object [13]. However, as our recent review has shown, no studies combined object position measurement with task-specific strength measurement during performance of UE ADL tasks [14]. We chose to focus on children with USCP because they often have clear problems using both hands together. They usually have less strength and control in the affected hand and often prefer to use only their non-affected hand. This leads to reduced bimanual functional performance. USCP is also a common condition in pediatric rehabilitation. These reasons make this group especially important to study. Since there are no similar studies using typically developing children for this kind of task, we focused on USCP to better understand their specific challenges.

To include the measurement of object position during task-specific strength measurements, the Activity Daily Living Test and Training Device (ADL-TTD) was developed. Reliability and validity of task-oriented strength testing, including object positioning, are not yet known, indicating a need for reliability and validity research performed in accordance with the COSMIN (COnsensus-based Standards for the Selection of Health Measurement INstruments) guideline [15], which also states that test–retest reliability should be tested before validity.

This study investigated test–retest reliability of MVC and object position in a bimanual box lifting task with “ADL-TTD” in children with USCP, while validity will be tested in a subsequent study.

## 2. Methods

### 2.1. Design

Children were recruited from the ‘Adelante’ rehabilitation center, the Hague and Leiden locations of the ‘Basalt’ rehabilitation center, the ‘Revant’ rehabilitation center in Breda, and ‘Revalidatie Friesland’ in the Netherlands. The following inclusion and exclusion criteria were applied.

#### 2.1.1. Inclusion Criteria

- Age between 7 and 18 years.
- Diagnosed with USCP by a pediatric neurologist.
- Gross Motor Function Classification System (GMFCS) level I–III [16].
- Manual Ability Classification System (MACS) level I–III [17].
- Manual function limitation classified as Zancolli degree I–IIb [18].
- Able to understand the test instructions and all attended regular primary or secondary school.
- Dutch speaking.

#### 2.1.2. Exclusion Criteria

- Botulinum Toxin-A injections in one or both upper extremities within the past three months.
- UE surgery within the past six months.

#### 2.1.3. Sample Size

The sample size is based on the COSMIN guideline for reliability studies, being minimally 50 participants. Although 47 participants were initially recruited, 4 were excluded due to technical problems with the motor or IMU, which prevented complete data collection. These participants were excluded from all reliability analyses.

The study was approved by the Medical Ethics Committee of Maastricht University with the Protocol ID: NL201803499/20110. The children were selected by the treatment teams at each center and informed about the study. After being informed adequately about the study and receiving informed consent from either by child and parents (12 years and older) or the carers for children younger than 12 years, the children were invited for inclusion assessments.

### 2.2. Devices

**Figure 1:**
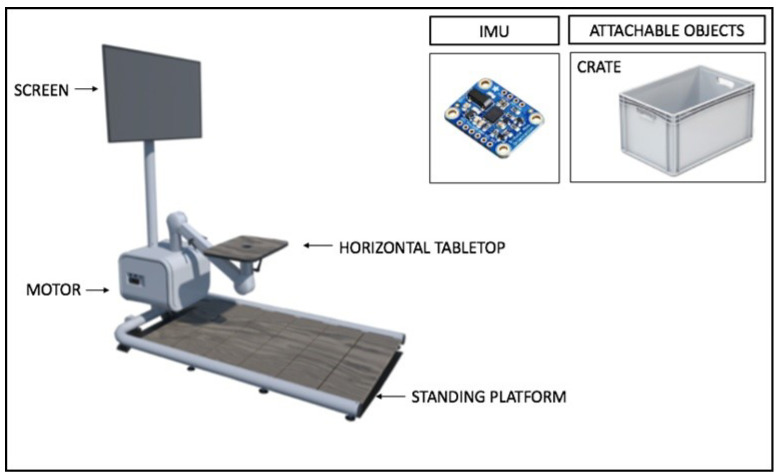
The parts of an ADL-TTD.

The ADL-TTD is a multifunctional device developed by UMACO B.V. in Groningen, the University of Maastricht, and the Adelante rehabilitation center and funded by the Johanna Kinderfonds and Health Holland. The device includes a motor, an IMU sensor (Adafruit BNO055 Absolute Orientation Sensor), a horizontal desktop, and attachable objects such as a plate, a box (for bimanual tasks), or a cup (for unimanual tasks). The materials are shown in Figure 1. The objects are connected to the device by a cable. The motor has the properties of an ergometer, so when the object is pulled, the metal cable will also be rolled out and locked at a specific height, enabling the measuring unit to determine the object’s isometric force.

The IMU sensor was affixed to the center of the box using Velcro. An IMU sensor has three sensor types: an accelerometer, a gyroscope, and a magnetometer. An IMU sensor can precisely measure acceleration, angular velocity, and magnetic field by integrating the data collected from various sensors. The ADL-TTD uses the IMU to measure the position of the associated object’s movement. This is measured in degrees (°) of tilt along the x, y, and z axes. The deviations around the x-axis are the anterior tilt (A-tilt) and posterior tilt (P-tilt), those around the y-axis are clockwise rotation (CL-rotation) and anticlockwise rotation (A-CL rotation), and those around the z-axis are AH downward tilt and NAH downward tilt. The ADL-TTD is linked to a laptop with the necessary software installed (ADL-TTD analyzer). The application illustrates the position measured in degrees, the height of the item from the tabletop, and the maximum isometric force. The specifications regarding the ADL-TTD and the Adafruit BNO055 Absolute Orientation Sensor are shown in Table A1 and Table A2.

### 2.3. Measurement Procedure

**Figure 2:**
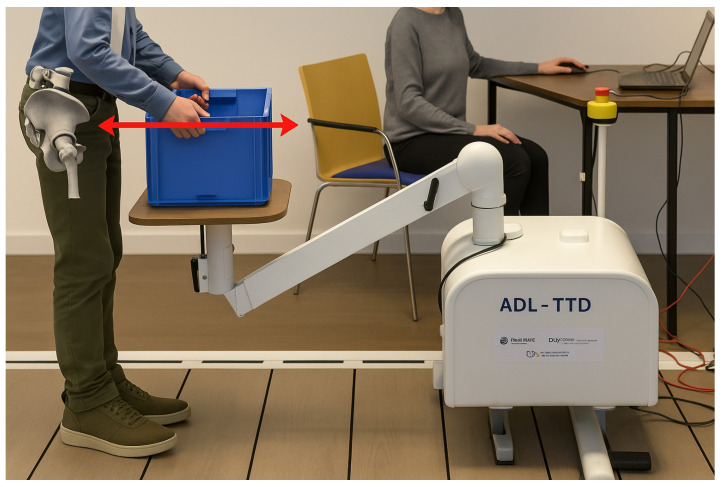
Measurement position when using ADL-TTD. Note: the red arrow means the hight of the box.

**Figure 3:**
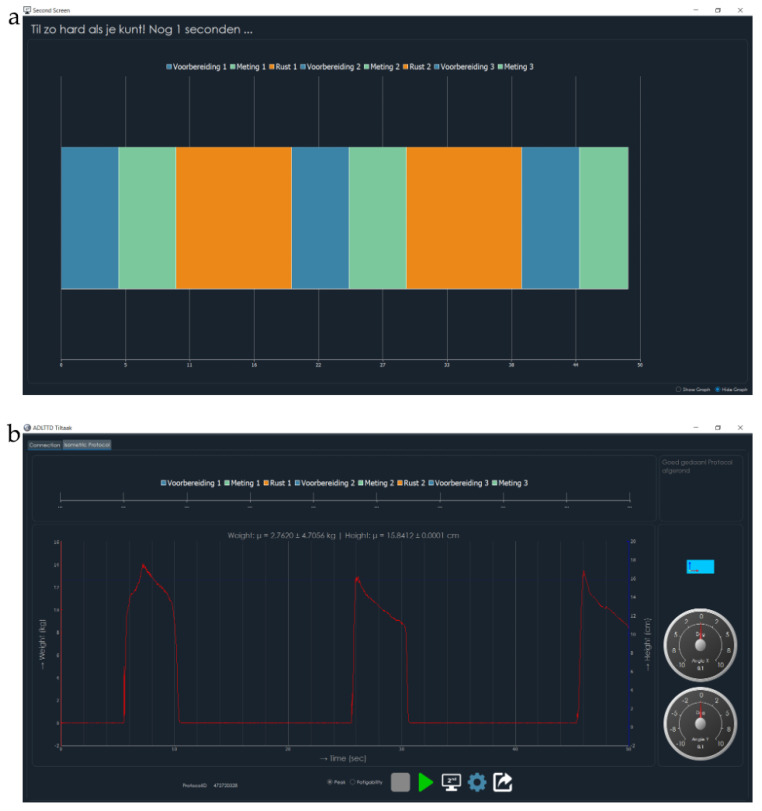
Screens shown during the test. (**a**) Display seen by the participant. Blue time zone: preparation time of five seconds. Green time zone: measurement time of five seconds. Orange time zone: recovery time of ten seconds (partially shown). (**b**) Display seen by the tester: an overview of two maximum force measurements. The blue line shows object height from the tabletop; the red line shows applied force over time.

The measurement procedure using ADL-TTD involves a series of steps to ensure that the device is properly calibrated and set up for use. Each participant stands in front of the device. The box is then attached to the cable and placed on a horizontal tabletop at the level of the participant’s pelvis (shown in Figure 2). The box could be lifted a maximum of 15 cm from the desktop, limited by the cable’s length. When the cable is pulled, the isometric strength is measured. The device is then recalibrated in this position. A screen visible to the participant displayed time zones in various colors, indicating different phases of the measurement process (Figure 3a). The blue color indicates a 5 s preparation time, during which the participant is required to lift the box slowly to the maximal height from the table. The green color represents a task period of five seconds, during which the participant is instructed to exert maximum effort to lift the box while keeping the box stable while standing without leaning backward, forwards, or sidewards. The orange color signifies a 10 s relaxation period, during which the participant can place the box on the tabletop and rest. The measurement is repeated three times while being monitored by the tester. The second screen (Figure 3b) shows the performance of the child. To avoid interference, this cannot be seen by the participant.

The testing process for each participant utilized Hebert’s protocol, as described for the HHD measurement, to enhance testing stability and guarantee maximum strength in every trial [19]. The Hebert protocol describes that when MVC varied by more than 20% across the three measurements, up to two additional trials should be conducted following the same protocol. Only the measurements within a 20% variance were considered valid. Three measurements within the range of 20% difference were selected. Meanwhile, during the whole period of lifting the position data of the box were also captured by IMU. Each participant underwent this testing procedure twice—test (T0) and retest (T1)—with an intersession interval ranging from 60 min to 24 h. All the raw data were documented after each test.

### 2.4. Procedure for Scoring the Outcome

The first outcome indicator for this evaluation is the MVC measured in kilograms, for which the average of three MVC measurements that fall within the 20% limit of difference is calculated.

**Figure 4:**
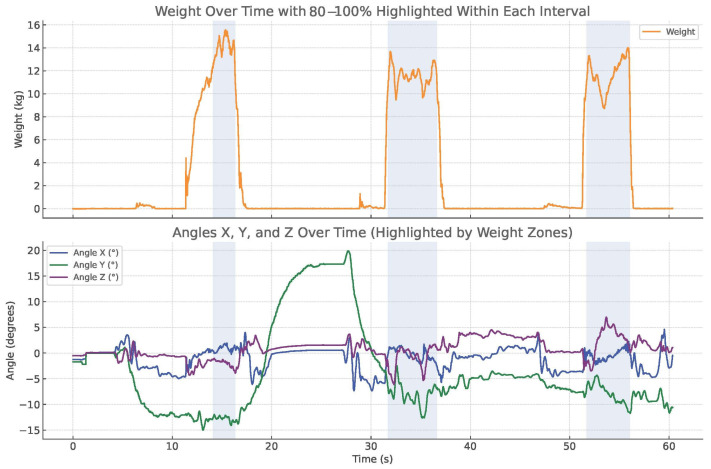
Applied force and X, Y, Z angles registered by ADL-TTD of three attempts.

**Figure 5:**
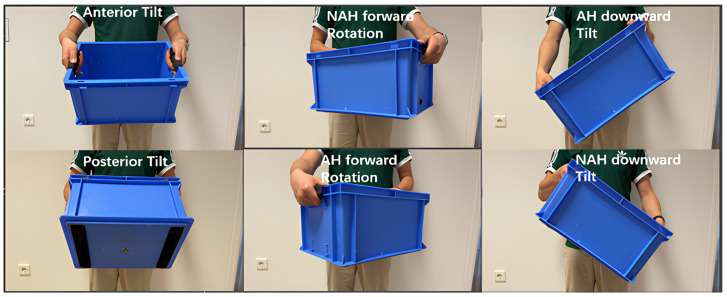
Orientation of the box based on the affected side (AH: affected hand, NAH: Non-affected hand; the right hand is the AH).

The second outcome is the mean position of the box relative to the x, y, and z axis, measured in degrees, using an IMU in the maximal zone. Figure 4 shows the graphs of strength and the x, y, and z angles against time. The maximal zone is defined as the zone from 80 to 100% of the MVC, in which maximal contraction of the muscle fibers occurs. The starting position of the box, and thus the IMU, was established as the tabletop of the ADL-TTD, which served as the reference or 0-position. Throughout the measurement, including the lifting, MVC, and repositioning of the box on the tabletop, the IMU continuously monitored the movement of the box about the three axes. During the lifting task, six directions of movement were recorded using the IMU: anterior/posterior tilt, forward rotation on both the affected and non-affected sides, and downward tilt on both sides. We chose to focus our analysis on three directions—posterior tilt, forward rotation on the non-affected side, and downward tilt on the affected side—because these were most commonly seen in clinical practice and during pilot testing. This decision was based on expert opinion from pediatric rehabilitation clinicians and an initial visual inspection of the full dataset. The other three directions were also recorded but are not included in the current analysis. We plan to explore them in future studies (shown in Figure 5).

In this study, the focus was the deviation of the box from the reference position during maximal isometric force production. The raw data obtained from the ADL-TTD assessment were uploaded into a dedicated application called “ADL-TTD Analyzer”, which was designed collaboratively by Adelante and UMACO. During the analysis, the application allowed the rater to select the three MVC values according to the graph display of the ADL-TTD analyzer. For each MVC, the rater was required to identify the corresponding time interval falling within the maximal zone. Following the selection of the maximal zones, the rater proceeded to calculate the mean values for the anterior/posterior tilt, forward movement on the unaffected/affected side, and affected/unaffected tilt during these time intervals. All the resulting values were recorded in an Excel spreadsheet, ensuring systematic data organization. All data were scored by one trained rater who was not involved in the testing sessions. The scoring was performed using a standardized manual developed during pilot testing to guide the identification of MVC intervals and positional data. To ensure consistency, intra-rater reliability was assessed during a pilot test, where the same rater analyzed a subset of recordings twice. The results showed good agreement, supporting the reliability of the scoring process. These steps were taken to reduce variability and potential bias in the data analysis.

### 2.5. Statistical Analysis

#### 2.5.1. Descriptive Statistics of the Population

In this study, we provide a clear understanding of the participant characteristics by reporting the demographics and descriptive statistics of the sample population. We summarize these by presenting the number of participants, sex distribution, mean age (with SD), and affected hand (Right/Left). Additionally, we describe the distribution across GMFCS, MACS, and Zancolli classification levels.

#### 2.5.2. ICC and SEM

To explore test–retest reliability of MVC and the position of the object in 3D space, we first investigated the intraclass correlation coefficients (ICC), confidence intervals (CI), and standard error of measurement (SEM) of the raw data.

The ICC(2,1) two-way random effects model with absolute agreement and a 95% confidence interval was used to determine relative reliability, following the guidelines of Koo and Li [20]. According to their interpretation, ICC values below 0.5 indicate poor reliability, values between 0.5 and 0.75 indicate moderate reliability, values between 0.75 and 0.9 indicate good reliability, and values above 0.9 indicate excellent reliability.

The SEM_agreement_ was calculated using the following formula:

$${\mathrm{SEM}}_{\mathrm{agreement}}=\sqrt{\sigma_{o}^{2}+\sigma_{\mathrm{residual}}^{2}}$$

where $\sigma_{o}^{2}$ represents the variance due to systematic differences between time points and $\sigma_{\mathrm{residual}}^{2}$ represents the random error variance. The SEM_agreement_ provides an estimate of how repeated measurements of an individual using the same instrument tend to be distributed around the “true” score, and it is reported as an absolute value.

#### 2.5.3. Absolute Agreement with Bland–Altman Plot

Bland–Altman plots with limits of agreement (LOA) were assessed to investigate absolute agreement between the test and retest measurement. LOA were determined as the mean ± (1.96 × SD) of the difference between the two test measurements. In a Bland–Altman plot, the difference between two measurements is plotted against their average, with the solid middle line showing the average difference. The upper and lower dashed lines represent the calculated LOA, mean difference ± 1.96 standard deviations). Statistical analyses were performed using SPSS Statistics 26 (IBM SPSS Statistics 26, ©IBM Armonk, NY, USA) and MedCalc for Windows, version 19.4 (MedCalc Software, Ostend, Belgium).

We tested the assumptions required for ICC and Bland–Altman analysis. Normality of the differences was assessed using the Shapiro–Wilk test. Homoscedasticity was evaluated by visually inspecting residual plots. No clear violations of these assumptions were found.

## 3. Results

### Descriptives

A total of 47 participants were recruited, of whom 43 had complete data for both sessions (tests and retests) and were included in the analysis. To assess potential bias, we compared the included and excluded participants on age, sex, affected side, and MACS and GMFCS levels. No relevant differences were observed between the two groups. Table 1 shows a detailed description of the participants. One tester was responsible for conducting the tests and another, the blinded tester, which was not involved in testing, was in charge of scoring.

Based on clinical observations, children show a preference for movements that involve posterior tilt, forward rotation on the non-affected hand, and downward tilt on the affected hand. So, we decided to focus our reliability analysis on these three positional parameters.

The first aim of the study was to calculate the ICC_agreement_ and SEM_agreement_. The mean MVC and positional values at T0 and T1 are presented in Table 2. The ICC_agreement_ for the mean MVC value was 0.95 (95% CI = 0.91 to 0.97), indicating excellent reliability. The SEM_agreement_ for the mean MVC value was 5.94 kg (Table 3). The ICC_agreement_ for the mean posterior tilt was 0.82 (95% CI = 0.66 to 0.90), indicating good reliability, and the SEM_agreement_ was 1.48 degrees. The ICC_agreement_ for the mean forward movement on the affected side was 0.83 (95% CI = 0.69 to 0.91), also indicating good reliability. The SEM_agreement_ was 5.39 degrees. The ICC_agreement_ for the mean affected-side tilt was 0.87 (95% CI = 0.75 to 0.93), showing good reliability. The SEM_agreement_ was 3.43 degrees. To aid clinical interpretation, the percentage SEM (SEM%) and minimal detectable change at 95% confidence (MDC_95_) were also calculated for each parameter. The SEM% for MVC was 57.9%, and the MDC_95_ was 16.45 kg, calculated using the formula 1.96 × $\sqrt{2}$ × SEM. For object positioning outcomes, the MDC_95_ was 1.99° for posterior tilt, 14.91° for NAH forward rotation, and 9.41° for AH downward tilt.

**Figure 6:**
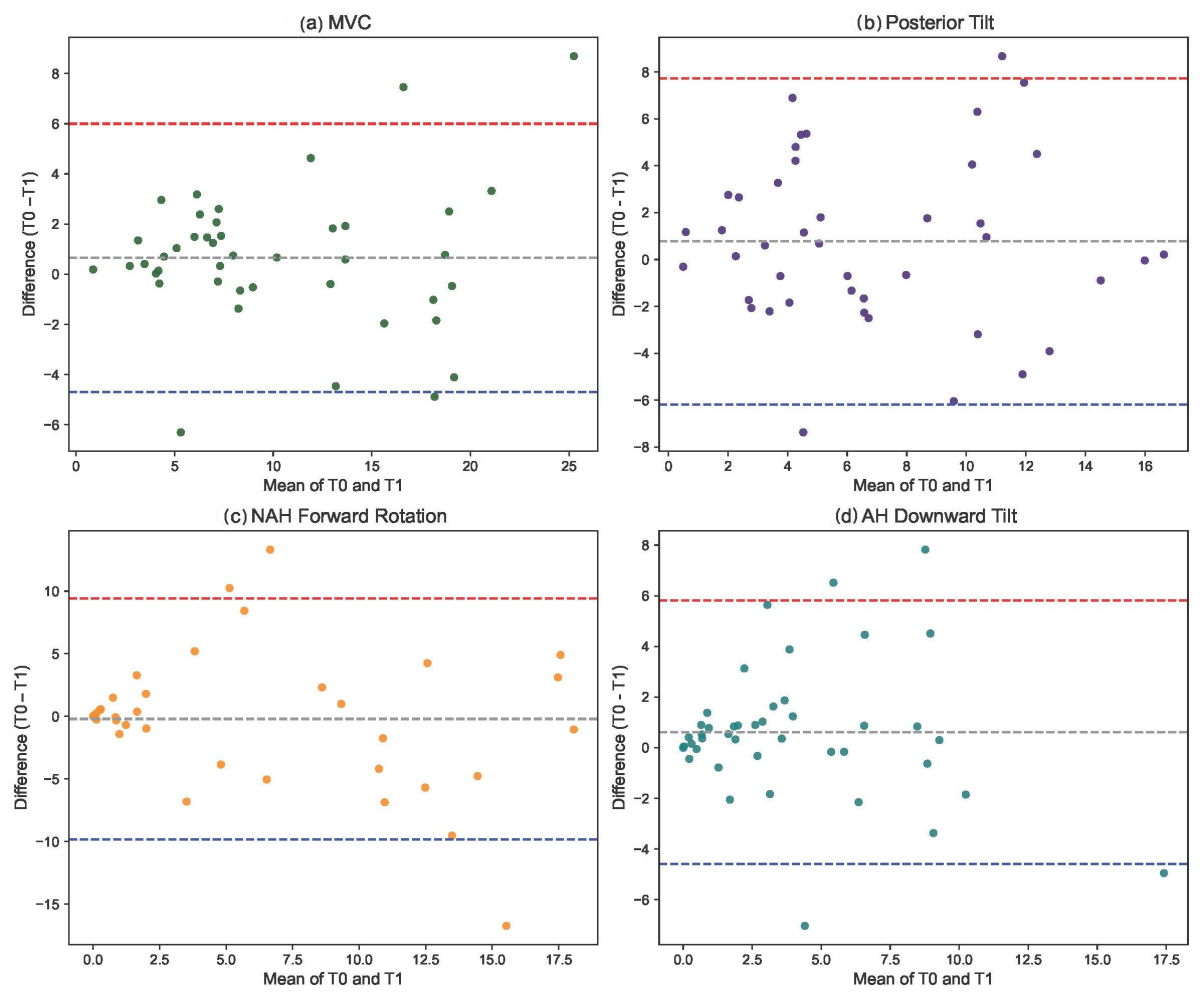
Bland–Altman plot based on original data. (**a**) MVC value; (**b**) posterior tilt; (**c**) NAH forward rotation; (**d**) AH downward tilt).

The absolute agreement measured with the Bland–Altman plots is shown in Figure 6. The mean differences between the two measurements and the LOA are shown. Outliers were also identified using the Bland–Altman plot. The presence of no more than three outliers in each plot can suggest that the agreement between the two measurements is generally good for most of the data.

## 4. Discussion

We examined the test–retest reliability of MVC and the position of the object in 3D space within the bimanual box task, assessed with the ADL-TTD in children with USCP. The results show very good test–retest reliability on MVC strength and good reliability regarding the position of posterior tilt, forward movement on the affected side, and downward tilt on the affected side. This is the first time the object position has been combined with the MVC while children with USCP performed ADL tasks. We used the IMU to identify deviations in the movement of the object in three dimensions during the lifting task.

### 4.1. Reliability in MVC

The mean MVC, with an ICC_agreement_ of 0.94, falls into the “excellent” reliability category (ICC > 0.9). This implies that for the metric of the MVC value, the measurements were highly consistent between T0 and T1. Comparing MVC reliability results of the ADL-TTD with reliability results of the HHD, which is the standardized strength measurement most frequently used in UE strength measurement for children, one study used HHD to measure isometric muscular strength in children with typical development (ICC_agreement_ = 0.53–0.95) and showed fair to excellent reliability [21] while another study conducted HHD for UE MVC measurement in USCP children, showed excellent test–retest reliability (ICC_agreement_ = 0.88–0.96) [7]. The results of ADL-TTD are in line with previous research which both show good reliability. The impact of the 20% rules can impact the height of the ICC; however, the advantage for clinical use is the agreement about the acknowledgment of the variation in testing and decreasing this variation by consensus of the 20% to be more in line with the results of the testing [19]. Meanwhile, measurement error should also be considered. For the measurements to be useful in assessment over time, the measurement error should be small to effectively detect actual changes over time [22]. When compared with other task-oriented strength measurements of the UE, one study showed that the box-task—a functional test for hand and UE muscle strength in children aged 7 to 12 years with USCP—has excellent test–retest reliability (ICC_agreement_ = 0.93) [9], although the SEM_agreement_ of 1.38 kg (22.4%) with a mean MVC 6.16 kg is large. Another study [10] of Geijen showed good test–retest reliability of the MVC in an instrumented box lifting task for both the 6–12-year-old (ICC_agreement_ = 0.95), and 13–18-year-old groups (ICC_agreement_ = 0.84) in children with USCP. However, the SEM_agreement_ for both groups was 1.49 kg (31.1%) and 2.33 kg (22.9%), respectively. The SEM_agreement_ of the MVC with the ADL-TTD is 5.94 kg (57.9%), which is large but in line with previous research. This is crucial for clinical use in treatment before and after, to ensure that the MVC truly changed after treatment and the change is not caused by the natural variance between two measures; the strength needs to be bigger than this. Although the SEM agreement of the MVC with the ADL-TTD is 5.94 kg (57.9%), which aligns with earlier studies [10] of Geijen, the minimal detectable change (MDC_95_) of 16.45 kg highlights a limitation in using the device for tracking change over time. This means that only strength gains exceeding 16.45 kg can be confidently interpreted as true improvement beyond measurement error. Such a large threshold confirms that the current application of the ADL-TTD is best suited for diagnostic or cross-sectional use, rather than for monitoring individual treatment progress. This is consistent with previous literature on task-oriented strength assessments, and we have intentionally followed the way this issue was addressed by Melanie Geijen and colleagues in their earlier reliability studies.

### 4.2. Reliability in Object Position

The ICC_agreement_ values for the mean posterior tilt, mean forward movement on the affected side, and mean downward tilt on the affected side all have ICC_agreement_ values in the “good” reliability range (0.81–0.86). We focused on three directions of movement because these were the most frequently observed patterns during the task, both in pilot testing and in clinical experience. Posterior tilt, forward rotation on the non-affected side, and downward tilt on the affected side were consistently noticeable and relevant for interpreting children’s motor control during lifting. Although all six directions were recorded by the IMU, we chose to analyze only these three in this study to keep the focus on the most meaningful and reliable parameters. The remaining directions will be explored in future work. This is the first time an IMU has been used to measure the position of the object in degrees. IMU showed good to excellent reliability on gait analysis, cervical coupling motion, shoulder range of motion, etc. [23,24,25,26,27]. Previous research on IMU mostly focused on acceleration, velocity, or range of motion. For example, Antunes et al., using IMU to measure knee range of motion, reported an average inaccuracy of 4.2° [28]. Another study reported that the IMU system measures the orientation of each body part with an accuracy of about 3° [29]. Besides, we could not find any research focused on the reliability of IMU used in object positioning. The SEM_agreement_ of IMU in ADL-TTD for posterior tilt, forward movement, and downward tilt on the affected side are 0.71°, 5.39°, and 3.43°, respectively, which are somewhat smaller, but comparable with those two studies. The number of degrees (0.71–5.39°) of deviation in positioning a box is low, indicating that children with USCP can perform precise positioning in a bimanual lifting task. This exciting finding indicates that such a small measurement error could have widespread clinical applications. Not only does it provide more precise measurements, but it also allows for the detection of slight changes in a patient’s condition over time.

In summary, the ADL-TTD seems to be a reliable tool for measuring the MVC of UE and object position based on the good ICC_agreement_ for the study population over a maximum of 24 h. For the aspect of object position, it seems more accurate in tracking the position of objects. However, the large SEM_agreement_ values of MVC suggest that to ensure a true change has occurred, individual children need to demonstrate relatively large changes in their strength scores.

### 4.3. Strengths and Limitations

In this study, we innovatively combined MVC and object positioning during UE tasks in ADL, focusing particularly on children with USCP. This approach significantly contributes to understanding a person’s UE movement quality during lifting tasks. For the first time, IMU were used to describe object positioning, exhibiting excellent reliability. The findings indicate that we can use the measure for diagnostic purposes at a certain timepoint and indeed use it to adapt a treatment plan. For example, the ranges in object position detected by IMU can tailor task-oriented strength training, by adding limits in positions while lifting the object. The use for evaluation is less obvious due to the large SEM of the MVC measurement. A large change in MVC due to treatment is needed to show effect of treatment. This method, by providing a more detailed understanding of ADL functional measurement, aligns closely with the individualized needs of pediatric rehabilitation, making it a valuable tool for therapists in tailoring training for this specific population.

The time interval between the test and retest ranged from 60 min to 24 h due to scheduling constraints at different rehabilitation centers. While this may introduce some variability, all test–retest sessions were performed within the same day, and no therapy was provided between sessions. This helped minimize the risk of actual clinical changes influencing the results. The order of testing was not randomized or counterbalanced. However, all retests were performed after a break and in a rested state. Also, a different rater, who was blinded to the test order, performed the scoring. These steps were taken to reduce potential fatigue or learning effects during retesting. Instead of seeing this variation as just a drawback, it is important to recognize that it mirrors what often happens in actual clinical settings where assessment times are not always the same; however, we used this to prevent that the found variance could be attributed to the child’s development or effects of treatment provided. Our study sample was diverse, reflecting the population being treated within the Dutch rehabilitation settings. This diversity is important because it captures the variability found in real-world contexts, even though it may have had a beneficial impact on the ICC values, indicating more agreement. Therefore, it should not be considered a limitation but rather a strength that enhances the generalizability of our findings. We acknowledge that there may be variability in measuring results if children have differing age or MACS classifications. This strategy, however, is in line with the exploratory character of our research and offers a comprehensive understanding that can be applied to different pediatric rehabilitation populations.

The study’s most significant disadvantage is its small sample size which may have an impact on the study’s statistical power and generalizability. According to the guidelines, around 50 subjects are often recommended as a solid starting point to achieve reliable and valid results [30] We only have 43 subjects, partly due to the COVID period, which limited our ability to test more participants, and partly due to a technical problem that caused data loss. Another limitation is that the data analysis process currently requires manual effort from the tester, which can be time-consuming. To address these issues in the future, we could develop algorithms to facilitate data analysis. This would not only save time but also help minimize bias, leading to more accurate and reliable results.

## 5. Conclusions

It has been shown that the ADL-TTD has good test–retest reliability when measuring MVC of UE tailored object position in task-oriented bimanual box lifting tasks for children with USCP. A good ICC_agreement_ value implies that the device gives consistent outcomes of more measurements performed within a timeframe where the child is assumed stable regarding the measured construct. It would be a good tool for clinicians to analyze specific populations in a cross-sectional situation. On the other hand, high SEM_agreement_ indicates that there is an important amount of error or variability in the individual measurements. That means that clinicians who wish to use it as a clinical device for an individual cannot use the outcomes for measuring changes over time. An important application for daily life is that MVC testing combined with object position can provide patients and therapists with insight into how to safely perform lifting tasks such as carrying hot liquids or fragile items on a tray. For instance, patients with USCP on one side in their UE may need to be particularly careful when lifting objects like hot liquids or fragile items. Research can identify the safest techniques, such as the correct posture, optimal grip, and appropriate lifting speed, to minimize the risk of injury or accidents. Therapists can use insights from these studies to create customized rehabilitation programs for patients. For example, they can observe whether the MVC is lower or in which direction tilting occurs and address it by training specific muscles or using techniques to inhibit issues such as spasm or tension in antagonists. Future research may explore the reduction of tilting while using the same amount of force and measuring it to investigate whether children with USCP could improve their lifting performance.

## Figures and Tables

**Table 1:** Characteristics of participants (N = 43). Abbreviations: GMFCS = Gross Motor Function Classification System; MACS = Manual Ability Classification System.

Characteristic	Value		
Sex (Male/Female)	20 (46.5%)	23 (53.5%)	
Mean Age (SD)	12.5 (2.4)		
Affected Hand (Right/Left)	27 (62%)	16 (38%)	
GMFCS Level (I/II)	37 (86%)	6 (14%)	
MACS Level (I/II/III)	12 (28%)	26 (60%)	5 (12%)
Zancolli Grade (1/2a/2b)	26 (60%)	14 (33%)	3 (7%)

**Table 2:** Mean values and absolute differences between T0 and T1 (abbreviations, NAH: non-affected hand, AH: affected hand).

Parameters	Mean T0	Mean T1	Absolute Difference (SD)
MVC (kg)	10.58	9.93	0.65 (2.67)
Posterior tilt (°)	7.17	6.40	0.77 (3.47)
NAH forward rotation (°)	5.04	5.25	3.05 (4.76)
AH downward tilt (°)	4.30	3.69	1.81 (0.27)

**Table 3:** Intraclass correlation coefficient (ICC_agreement_), 95% confidence interval (CI), standard error of measurement (SEM_agreement_), percentage SEM (SEM%), and minimal detectable change (MDC_95_).

Parameter	ICC_agreement_	95% CI	SEM_agreement_	SEM%	MDC_95_
MVC (kg)	0.95	0.91 to 0.97	5.94 kg	57.9%	16.45 kg
Posterior tilt (°)	0.82	0.67 to 0.90	0.71	9.9%	1.99°
NAH forward rotation (°)	0.83	0.69 to 0.91	5.39	104.3%	14.91°
AH downward tilt (°)	0.86	0.75 to 0.91	3.43	79.8%	9.41°

## Data Availability

The raw data supporting the conclusions of this article will be made available by the authors on request due to privacy.

## Abbreviations

The following abbreviations are used in this manuscript:

USCP	Unilateral Spastic Cerebral Palsy
MVC	Maximum Voluntary Contraction
ICC	Intraclass Correlation Coefficient
CI	Confidence Interval
SEM	Standard Error of Measurement
ADL	Activity of Daily Living
IMU	Inertial Measurement Units
LOA	Limits of Agreement
AH	Affected Hand
NAH	Non-Affected Hand
ADL-TTD	Activity of Daily Living Testing and Training Device
TAAC	Task-oriented Arm–Hand Capacity
COSMIN	COnsensus-based Standards for the Selection of Health Measurement INstruments
SD	Standard Deviation
MACS	Manual Ability Classification System
GMFCS	Gross Motor Function Classification System
HHD	Hand-Held Dynamometry
MDC	Minimal Detectable Change
UE	Upper Extremity

## Appendix A

**Table A1:** Device specifications.

Specification	Value
Dimensions (L × W × H)	180 × 100 × 80 cm
Height adjustment range	60–120 cm (height of tabletop)
Weight of device	75 kg
Accuracy of lifting (height)	±2 mm
Accuracy of lifting (weight)	To lift 5 kg: ±10 g From 5 kg to 40 kg: ±0.5%
Maximum lifting height from tabletop	70 cm
Maximum lifting weight	40 kg
Power supply	240 V, 50/60 Hz

**Table A2:** Specifications of Adafruit BNO055 Absolute Orientation Sensor.

Measurement	Specifications
Absolute Orientation (Euler)	→ Euler Vector, 100 Hz Three-axis orientation data based on a 360° sphere
Absolute Orientation (Quaternion)	→ Quaternion, 100 Hz Four-point quaternion output for more accurate data manipulation
Angular Velocity Vector	→ 100 Hz Three-axis rotation speed in rad/s
Acceleration Vector	→ 100 Hz Three-axis acceleration (gravity + linear motion) in m/s^2^
Magnetic Field Strength Vector	→ 20 Hz Three-axis magnetic field sensing in microteslas (μT)
Linear Acceleration Vector	→ 100 Hz Three-axis linear acceleration data (acceleration minus gravity) in m/s^2^
Gravity Vector	→ 100 Hz Three-axis gravitational acceleration (minus any movement) in m/s^2^
Temperature	→ 1 Hz Ambient temperature in degrees Celsius
